# A Complex Immunological Idiotypic Network for Maintenance of Tolerance

**DOI:** 10.3389/fimmu.2014.00369

**Published:** 2014-07-31

**Authors:** Véronique Thomas-Vaslin

**Affiliations:** ^1^UPMC Univ Paris 06, UMRS959, Immunology-Immunopathology-Immunotherapy (I3), Paris, France; ^2^CNRS, FRE3632, Immunology-Immunopathology-Immunotherapy (I3), Paris, France; ^3^INSERM, UMRS959, Immunology-Immunopathology-Immunotherapy (I3), Paris, France

**Keywords:** tolerance mechanisms, idiotypic network, modeling and simulations, complex system, lymphocytes

The immune system is a multi-scale, self-assembling, adaptive, dynamic, and cognitive network of diverse interacting agents with high turnover, as cells and molecules. This system perceives the quality and the quantity of microscopic patterns as antigens, derived from the body or the environment and remembers them to define the temporal identity of the organism in the spatial scale (from molecule to organs), and within the changing micro/macroscopic environment. The repertoire of B and T lymphocytes represents the collection of diverse lymphocytes and their immuno-receptors, which develop from stochastic, somatic, DNA VDJ genes rearrangement and from the huge quantity of new lymphocytes that enter the network daily. By continuously “selfing,” the lymphocyte repertoire evolves from ontogeny (pure eukaryotic fetus) to aging influenced by the microbiota. This leads to decision-making for the survival/death/proliferation of individual heterogeneous cell clones (1), up to the collective behavior of the network, leading to tolerance or to an effective immune response. Thus, each lymphocyte is submitted to considerable selection to enter the “available repertoire” as complementary images of the cognate antigens, leading to internal activity in the central network (2). Lymphocyte activation leads to the selection of the clones involved in the cellular and/or humoral immune response. Consequently, the lymphocyte repertoire constantly varies in time and space as a dynamic internal image of the lymphocyte environment, being it of idiotypic, cellular, molecular, bacterial, viral, or chemical origin. A dynamic equilibrium of cellular and molecular networks is thus involved in the control of body integrity and identity, leading to a “dynamic self.” Self-cognition and auto-reactivity are therefore a physiologic behavior of 10–20% of lymphocytes (2), with regulation of natural circulating IgM actual repertoires (3) and represent the “concinnous” function of the immune system (4). The recognition of the internal/non-internal image of the immune-receptors leads to a potential “idiotypic network.” Therefore, lymphocyte dynamics, repertoires, and networks can be modeled in various forms (5–7) involving variable region selection by antigens for cell survival and expansion (8).

Schulz et al. (9) propose a minimal computational model of the spatio-temporal evolution of the idiotypic network and interactions of clones of B cells and Ig with or without self-interaction. Conceptually, in this model (Figure 1), representing the whole potential repertoire of immune-receptors, B cell clones can only survive in the network if each clone receives a deterministic signal through interactions and according to the affinity of the immuno-receptor for antigens. This signal should fall in an adequate window related to the frequency of other B cell clones and potential interaction with anti-idiotypes and/or other antigens. Filling the system with a continuous production from new random clones with diversified immune-receptors leads to progressive emergence of a network of relations within the clones, and equilibrium in the network is finally reached. At steady-state, according to the affinity of lymphocytes for antigens, their proliferation, and their life span, a given number of clones reach a given clone size and establish relations with other lymphocyte clones or idiotopes. The architecture of the network is determined according to the number of clones, their size, and connectivity structure. It remains stable for long periods of time if the influx of lymphocytes with a diversified VDJ repertoire is sustained. This situation might represent a physiological behavior.

**Figure 1:**
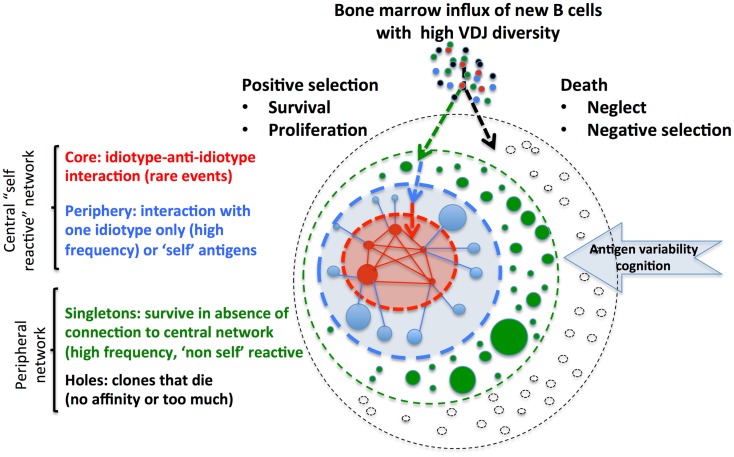
**Metadynamics and lymphocyte integration within the central idiotypic and peripheral network**. Permanent integration of new lymphocytes (exemplified for B cells) sustains receptor and idiotope diversity. Antigen interaction leads to a deterministic signal with positive selection and integration in network, or death. Size of the clone is represented by the size of the circle and links between clones by lines. Colors represent the potential interaction of B cell clones/antibodies with antigens and integration into the network. Introduction of self-antigen and cognition by B cells clones reinforce the central network that controls clone size and auto-reactivity. The central part of this network is represented by “self” reactive clones with high connectivity: rare “core” clones interact with corresponding anti-idiotypes clones and more frequent “periphery” clones that recognize other self-antigens. The peripheral part of the network is represented by very frequent “singletons,” which have no connectivity with other clones, but can potentially react with “non-self.” “Holes” in the potential network might appear when clones die, because they do not find link (no affinity) to antigens or because of negative selection or suppression. Similar systemic organization integrates T cells and recursive selection of lymphocyte repertoires (10).

Now, if auto-reactive clones (directed to self-antigens but not involved in anti-idiotypic reactions) that normally die because they received either no signal or too much and are represented here as “holes” are properly stimulated with self-antigens, this rescues them from cell death and they integrate the network as “singletons” or “periphery” B cell clones. At this time, the connectivity structure of the network is rapidly perturbed (the “old” singletons and periphery clones rapidly collapse and die because they lose connection). Progressively, a novel equilibrium is reached, integrating the new auto-reactive cells in the network as persisting clones, with a controlled size of the clones. This behavior provides indeed a state of tolerance: self-reactive clones are present, but their connectivity to highly connected (idiotype/anti-idiotype) clones regulates their size, limiting their auto-reactivity. This situation can mimic the perturbation related to infection, leading to cross reactivity to autoantigens, but finally autoimmunity could be controlled if the idiotype/anti-idiotype core is functional. This is reminiscent of the presence of anti-idiotype from autoantibodies during remission from autoimmune disease, or the remission induced by injection of purified serum whole Ig from cohorts of healthy patients (IVIG), supposed to contain idiotype/anti-idiotype immunoglobulins. A reduced influx would also decrease the complexity of the network and its resilience as observed with aging and increased frequency of autoimmunity. In the context of an infection and inflammation that could induce epitope spreading, molecular mimicry, or cryptic antigens exposure, autoimmune diseases might occur, resulting from a defect of immune response and lack of connection to the network (11). The observation of earlier kinetics of anti-idiotypic response to auto-antigen, before auto-antibody response, also suggests a role of rheumatoid factors recognizing idiotypes of antibodies, in regulation of auto-immunity (12).

Generally, the simulation of a complex network of interactions between highly diverse immune-receptor can help understanding the dynamic behavior, distributed memory (13), and the resilience of the complex immune system with interacting agents. Highly connected parts of the system (core) can regulate other less connected (peripheral) parts of the network. Dominant tolerance by regulation and control of clonal size of potential harmful lymphocytes is thus activated by self-cognition. Likewise, T lymphocytes positively selected on thymic medulla epithelial cells, presenting tissue specific antigens, form a ‘core’ in the global T lymphocyte network, with a repertoire biased to self-cognition and behave as natural regulatory T cells involved in dominant and tissue specific tolerance (14), while presentation of B cell idiotopes is required for full tolerance (15). Immunization conditions and antigen doses are expected to lead to immune, tolerant, or hyper-reactivity conditions (12). Yet, *in vivo* perturbation and shrinking of the network through aging or transient depletion of dividing lymphocytes affect repertoire composition (16), tolerance (17), and memory maintenance leading to immunological amnesia (18). Therapies for regulation of inflammation by ‘T cell vaccination’ or homuncular self-antigen immunization, by inducing a regulatory anti-idiotypic network, are thus proposed to cure auto-immune diseases in the long run rather than global immunosuppression (19). Otherwise, low-dose IL-2 administration to stimulate autoreactive Treg to control effector lymphocyte expansion during aging (16) or to prevent autoimmune diseases is promising.

## Conflict of Interest Statement

The author declares that the research was conducted in the absence of any commercial or financial relationships that could be construed as a potential conflict of interest.
